# A linear programming approach for estimating the structure of a sparse linear genetic network from transcript profiling data

**DOI:** 10.1186/1748-7188-4-5

**Published:** 2009-02-24

**Authors:** Sahely Bhadra, Chiranjib Bhattacharyya, Nagasuma R Chandra, I Saira Mian

**Affiliations:** 1 Department of Computer Science and Automation, Indian Institute of Science, Bangalore, Karnataka, India; 2 Bioinformatics Centre, Indian Institute of Science, Bangalore, Karnataka, India; 3 Life Sciences Division, Lawrence Berkeley National Laboratory, Berkeley, California 94720, USA

## Abstract

**Background:**

A genetic network can be represented as a directed graph in which a node corresponds to a gene and a directed edge specifies the direction of influence of one gene on another. The reconstruction of such networks from transcript profiling data remains an important yet challenging endeavor. A transcript profile specifies the abundances of many genes in a biological sample of interest. Prevailing strategies for learning the structure of a genetic network from high-dimensional transcript profiling data assume sparsity and linearity. Many methods consider relatively small directed graphs, inferring graphs with up to a few hundred nodes. This work examines large undirected graphs representations of genetic networks, graphs with many thousands of nodes where an undirected edge between two nodes does not indicate the direction of influence, and the problem of estimating the structure of such a sparse linear genetic network (SLGN) from transcript profiling data.

**Results:**

The structure learning task is cast as a sparse linear regression problem which is then posed as a LASSO (*l*_1_-constrained fitting) problem and solved finally by formulating a Linear Program (LP). A bound on the Generalization Error of this approach is given in terms of the Leave-One-Out Error. The accuracy and utility of LP-SLGNs is assessed quantitatively and qualitatively using simulated and real data. The Dialogue for Reverse Engineering Assessments and Methods (DREAM) initiative provides gold standard data sets and evaluation metrics that enable and facilitate the comparison of algorithms for deducing the structure of networks. The structures of LP-SLGNs estimated from the INSILICO1, INSILICO2 and INSILICO3 simulated DREAM2 data sets are comparable to those proposed by the first and/or second ranked teams in the DREAM2 competition. The structures of LP-SLGNs estimated from two published *Saccharomyces cerevisae *cell cycle transcript profiling data sets capture known regulatory associations. In each *S. cerevisiae *LP-SLGN, the number of nodes with a particular degree follows an approximate power law suggesting that its degree distributions is similar to that observed in real-world networks. Inspection of these LP-SLGNs suggests biological hypotheses amenable to experimental verification.

**Conclusion:**

A statistically robust and computationally efficient LP-based method for estimating the topology of a large sparse undirected graph from high-dimensional data yields representations of genetic networks that are biologically plausible and useful abstractions of the structures of real genetic networks. Analysis of the statistical and topological properties of learned LP-SLGNs may have practical value; for example, genes with high random walk betweenness, a measure of the centrality of a node in a graph, are good candidates for intervention studies and hence integrated computational – experimental investigations designed to infer more realistic and sophisticated probabilistic directed graphical model representations of genetic networks. The LP-based solutions of the sparse linear regression problem described here may provide a method for learning the structure of transcription factor networks from transcript profiling and transcription factor binding motif data.

## Background

Understanding the dynamic organization and function of networks involving molecules such as transcripts and proteins is important for many areas of biology. The ready availability of high-dimensional data sets generated using high-throughput molecular profiling technologies has stimulated research into mathematical, statistical, and probabilistic models of networks. For example, GEO [1] and ArrayExpress [2] are public repositories of well-annotated and curated transcript profiling data from diverse species and varied phenomena obtained using different platforms and technologies.

A genetic network can be represented as a graph consisting of a set of nodes and a set of edges. A node corresponds to a gene (transcript) and an edge between two nodes denotes an interaction between the connected genes that may be linear or non-linear. In a directed graph, the oriented edge *A *→ *B *signifies that gene *A *influences gene *B*. In an undirected graph, the un-oriented edge *A *- *B *encodes a symmetric relationship and signifies that genes *A *and *B *may be co-expressed, co-regulated, interact or share some other common property. Empirical observations indicate that most genes are regulated by a small number of other genes, usually fewer than ten [3-5]. Hence, a genetic network can be viewed as a sparse graph, *i.e*., a graph in which a node is connected to a handful of other nodes. If directed (acyclic) graphs or undirected graphs are imbued with probabilities, the result is probabilistic directed graphical models and probabilistic undirected graphical models respectively [6].

Extant approaches for deducing the structure of genetic networks from transcript profiling data [7-9] include Boolean networks [10-14], linear models [15-18], neural networks [19], differential equations [20], pairwise mutual information [10,21-23], Gaussian graphical models [24,25], heuristic approachs [26,27], and co-expression clustering [16,28]. Theoretical studies of sample complexity indicate that although sparse directed acyclic graphs or Boolean networks could be learned, inference would be problematic because in current data sets, the number of variables (genes) far exceedes the number of observations (transcript profiles) [12,14,25]. Although probabilistic graphical models provide a powerful framework for representing, modeling, exploring, and making inferences about genetic networks, there remain many challenges in learning *tabula rasa *the topology and probability parameters of large, directed (acyclic) probabilistic graphical models from uncertain, high-dimensional transcript profiling data [7,25,29-33]. Dynamic programing approaches [26,27] use Singular Value Decomposition (SVD) to pre-process the data and heuristics to determine stopping criteria. These methods have high computational complexity and yield approximate solutions.

This work focuses on a plausible, albeit incomplete representation of a genetic network – a sparse undirected graph – and the task of estimating the structure of such a network from high-dimensional transcript profiling data. Since the degree of every node in a sparse graph is small, the model embodies the biological notion that a gene is regulated by only a few other genes. An undirected edge indicates that although the expression levels of two connected genes are related, the direction of influence is not specified. The final simplification is that of restricting the type of interaction that can occur between two genes to a single class, namely a linear relationship. This particular representation of a genetic network is termed a sparse linear genetic network (SLGN).

Here, the task of learning the structure of a SLGN is equated with that of solving a collection of sparse linear regression problems, one for each gene in a network (node in the graph). Each linear regression problem is posed as a LASSO (*l*_1_-constrained fitting) problem [34] that is solved by formulating a Linear Program (LP). A virtue of this LP-based approach is that the use of the Huber loss function reduces the impact of variation in the training data on the weight vector that is estimated by regression analysis. This feature is of practical importance because technical noise arising from the transcript profiling platform used coupled with the stochastic nature of gene expression [35] leads to variation in measured abundance values. Thus, the ability to estimate parameters in a robust manner should increase confidence in the structure of an LP-SLGN estimated from noisy transcript profiles. An additional benefit of the approach is that the LP formulations can be solved quickly and efficiently using widely available software and tools capable of solving LPs involving tens of thousands of variables and constraints on a desktop computer.

Two different LP formulations are proposed: one based on a positive class of linear functions and the other on a general class of linear functions. The accuracy of this LP-based approach for deducing the structure of networks is assessed statistically using gold standard data and evaluation metrics from the Dialogue for Reverse Engineering Assessments and Methods (DREAM) initiative [36]. The LP-based approach compares favourably with algorithms proposed by the top two ranked teams in the DREAM2 competition. The practical utility of LP-SLGNs is examined by estimating and analyzing network models from two published *Saccharomyces cerevisiae *transcript profiling data sets [37] (ALPHA; CDC15). The node degree distributions of the learned *S. cerevisiae *LP-SLGNs, undirected graphs with many hundreds of nodes and thousands of edges, follow approximate power laws, a feature observed in real biological networks. Inspection of these LP-SLGNs from a biological perspective suggests they capture known regulatory associations and thus provide plausible and useful approximations of real genetic networks.

## Methods

### Genetic network: sparse linear undirected graph representation

A genetic network can be viewed as an undirected graph, G = {**V**, **W**}, where **V **is a set of *N *nodes (one for each gene in the network), and **W **is an *N *× *N *connectivity matrix encoding the set of edges. The (*i*, *j*)^*th *^element of the matrix **W **specifies whether nodes *i *and *j *do (*W*_*ij *_≠ 0) or do not (*W*_*ij *_= 0) influence each other. The degree of node *n*, *k*_*n*_, indicates the number of other nodes connected to *n *and is equivalent to the number of non-zero elements in row *n *of **W**. In real genetic networks, a gene is regulated often by a small number of other genes [3,4] so a reasonable representation of a network is a sparse graph. A sparse graph is a graph G parametrized by a sparse matrix **W**, a matrix with few non-zero elements *W*_*ij*_, and where most nodes have a small degree, *k*_*n *_< 10.

#### Linear interaction model: static and dynamic settings

If the relationship between two genes is restricted to the class of linear models, the abundance value of a gene is treated as a weighted sum of the abundance values of other genes. A high-dimensional transcript profile is a vector of abundance values for *N *genes. An *N *× *T *matrix **E **is the concatenation of *T *profiles, [**e**(1),..., **e**(*T*)], where **e**(*t*) = [*e*_1_(*t*),..., *e*_*N*_(*t*)]^⊤ ^and *e*_*n*_(*t*) is the abundance of gene *n *in profile *t*. In most extant profiling studies, the number of transcripts monitored exceeds the number of available profiles (*N *≫ *T*).

In the static setting, the *T *transcript profiles in the data matrix **E **are assumed to be unrelated and so independent of one another. In the linear interaction model, the abundance value of a gene is treated as a weighted sum of the abundance values of all genes in the same profile,

(1) $$\begin{array}{lll} e_{n}(t) & = & \sum_{j=1}^{N}w_{nj}e_{j}(t)\\ & = & w_{n}^{\text{T}}e(t)\\ & & \text{where }w_{nn}=0 \end{array}$$

The parameter **w**_*n *_= [*w*_*n*1_,..., *w*_*nN*_]^⊤ ^is a weight vector for gene *n *and the *j*^*th *^element indicates whether genes *n *and *j *do (*w*_*nj *_≠ 0) or do not (*w*_*nj *_= 0) influence each other. The constraint *w*_*nn *_= 0 prevents gene n from influencing itself at the same instant so its abundance is a function of the abundances of the remaining *N *- 1 genes in the same profile.

In the dynamic setting, the *T *transcript profiles in **E **are assumed to form a time series. In the linear interaction model, the abundance value of a gene at time *t *is treated as a weighted sum of the abundance values of all genes in the profile from the previous time point, *t *- 1, *i.e*., $e_{n}(t)=w_{n}^{\text{T}}e(t-1)$. There is no constraint *w*_*nn *_= 0 because a gene can influence its own abundance at the next time point.

As described in detail below, the SLGN structure learning problem involves solving *N *independent sparse linear regression problems, one for each node in the graph (gene in the network), such that every weight vector **w**_*n *_is sparse. The sparse linear regression problem is cast as an LP and uses a loss function which ensures that the weight vector is resilient to small changes in the training data. Two LPs are formulated and each formulation contains one user-defined parameter, *A*, the upper bound of the *l*_1 _norm of the weight vector. One LP is based on a general class of linear functions. The other LP formulation is based on a positive class of linear functions and yields an LP with fewer variables than the first.

### Simulated and real data

#### DREAM2 In-Silico-Network Challenges data

A component of Challenge 4 of the DREAM2 competition [38] is predicting the connectivity of three *in silico *networks generated using simulations of biological interactions. Each DREAM2 data set includes time courses (trajectories) of the network recovering from several external perturbations. The INSILICO1 data were produced from a gene network with 50 genes where the rate of synthesis of the mRNA of each gene is affected by the mRNA levels of other genes; there are 23 different perturbations and 26 time points for each perturbation. The INSILICO2 data are similar to INSILICO1 but the topology of the 50-gene network is qualitatively different. The INSILICO3 data were produced from a full *in silico *biochemical network that had 16 metabolites, 23 proteins and 20 genes (mRNA concentrations); there are 22 different perturbations and 26 time points for each perturbation. Since the LP-based method yields network models in the form of undirected graphs, the data were used to make predictions in the DREAM2 competition category UNDIRECTED-UNSIGNED. Thus, the simulated data sets used to estimate LP-SLGNs are an *N *= 50 × *T *= 26 matrix (INSILICO1), an *N *= 50 × *T *= 26 matrix (INSILICO2), and an *N *= 59 × *T *= 26 matrix (INSILICO3).

#### S. cerevisiae transcript profiling data

A published study of *S. cerevisiae *monitored 2,467 genes at various time points and under different conditions [37]. In the investigations designated ALPHA and CDC15, measurements were made over *T *= 15 and *T *= 18 time points respectively. Here, a gene was retained only if an abundance measurement was present in all 33 profiles. Only 605 genes met this criterion of no missing values and these data were not processed any further. Thus, the real transcript profiling data sets used to estimate LP-SLGNs are an *N *= 605 × *T *= 15 matrix (ALPHA) and an *N *= 605 × *T *= 18 matrix (CDC15).

#### Training data for regression analysis

A training set for regression analysis, ${\left\{D_{n}\right\}}_{n=1}^{N}$, is created by generating training points for each gene from the data matrix **E**. For gene *n*, the training points are $D_{n}={\left\{(x_{ni},y_{ni})\right\}}_{i=1}^{I}$. The *i*^*th *^training point consists of an "input" vector, **x**_*ni *_= [*x*_1*i*_,..., *x*_*Ni*_] (abundances values for *N *genes), and an "output" scalar *y*_*ni *_= *x*_*ni *_(abundance value for gene *n*).

In the static setting, *I *= *T *training points are created because both the input and output are generated from the same profile; the linear interaction model (Equation 1) includes the constraint *w*_*nn *_= 0. If *e*_*n*_(*t*) is the abundance of gene *n *in profile *t*, the *i*^*th *^training point is **x**_*ni *_= **e**(*t*) = [*e*_1_(*t*),..., *e*_*N*_(*t*)], *y*_*ni *_= *e*_*n*_(*t*), and *t *= 1,..., *T*.

In the dynamic setting, *I *= *T *- 1 training points are created because the output is generated from the profile for a given time point whereas the input is generated from the profile for the previous time point; there is no constraint *w*_*nn *_= 0 in the linear interaction model. The *i*^*th *^training point is **x**_*ni *_= **e**(*t *- 1) = [*e*_1_(*t *- 1),..., *e*_*N*_(*t *- 1)], *y*_*ni *_= *e*_*n*_(*t*), and *t *= 2,..., *T*.

The results reported below are based on training data generated under a static setting so the constraint *w*_*nn *_= 0 is imposed.

### Notation

Let $R^{N}$ denote the *N*-dimensional Euclidean vector space and card(*A*) the cardinality of a set *A*. For a vector **x **= [*x*_1_,..., *x*_*N*_]^⊤ ^in this space, the *l*_2 _(Euclidean) norm is the square root of the sum of the squares of its elements, ${\left\|x\right\|}_{2}=\sqrt{\sum_{n=1}^{N}x_{n}^{2}}$; the *l*_1 _norm is the sum of the absolute values of its elements, ${\left\|x\right\|}_{1}=\sum_{n=1}^{N}\left|x_{n}\right|$; and the *l*_0 _norm is the total number of non-zero elements, ||**x**||_0 _= *card*({*n*|*x*_*n *_≠ 0; 1 ≤ *n *≤ *N*}). The term **x **≥ 0 signifies that every element of the vector is zero or positive, *x*_*n *_≥ 0, ∀*n *∈ {1,..., *N*}. The one- and zero-vectors are **1 **= [1_1_,..., 1_*N*_]^⊤ ^and **0 **= [0_1_,..., 0_*N*_]^⊤ ^respectively.

### Sparse linear regression: an LP-based formulation

Given a training set for gene *n*

(2) $$D_{n}=\{(x_{ni},y_{ni})|x_{ni}\in R^{N};y_{ni}\in R;i=1,...,I\}$$

the sparse linear regression problem is the task of inferring a sparse weight vector, **w**_*n*_, under the assumption that gene-gene interactions obey a linear model, *i.e*., the abundance of a gene *n*, *y*_*ni *_= *x*_*n*_, is a weighted sum of the abundances of other genes, $y_{ni}=w_{n}^{\text{T}}x_{ni}$.

### Sparse weight vector estimation

#### *l*_0 _norm minimization

The problem of learning the structure of an SLGN involves estimating a weight vector such that **w **best approximates *y *and most of elements of **w **are zero. Thus, one strategy for obtaining sparsity is to stipulate that **w **should have at most *k *non-zero elements, ||**w**||_0 _≤ *k*. The value of *k *is equivalent to the degree of the node so a biologically plausible constraint for a genetic network is ||**w**||_0 _≤ 10. Given a value of *k*, the number of possible choices of predictors that must be examined is ^*N*^*C*_*k*_. Since there are many genes (*N *is large) and each choice of predictor variables requires solving an optimization problem, learning a sparse weight vector using an *l*_0 _norm-based approach is prohibitive, even for small *k*. Furthermore, the problem is NP-hard [39] and cannot even be approximated in time $2^{\log^{1-\varepsilon }N}$ where ϵ is small positive quantity.

#### LASSO

A tractable approximation of the *l*_0 _norm is the *l*_1 _norm [40,41] (for other approximations see [42]). LASSO [34] uses an upper bound for the *l*_1 _norm of the weight vector, specified by a parameter *A*, and formulates the *l*_1 _norm minimization problem as follows,

$$\begin{array}{ll} \underset{w,v}{\text{minimize}} & \sum_{i=1}^{I}\left|v_{i}\right|\\ \text{subject to} & w^{\text{T}}x_{i}+v_{i}=y_{i}\\ & {\left\|w\right\|}_{1}\leq A. \end{array}$$

This formulation attempts to choose **w **such that it minimizes deviations between the predicted and the actual values of *y*. In particular, **w **is chosen to minimize the loss function $L(w)=\sum_{i=1}^{I}\left|w^{\text{T}}x_{i}-y_{i}\right|$. Here, "Empirical Error" is used as the loss function. The Empirical Error of a graph G is $\frac{1}{N}\sum_{n=1}^{N}Empirical_{error}(D_{n})$, where $Empirical_{error}(D_{n})=\frac{1}{I}\sum_{i=1}^{I}\left|y_{ni}-f(x_{ni};w_{n})\right|$. The user-defined parameter *A *controls the upper bound of the *l*_1 _norm of the weight vector and hence the trade-off between sparsity and accuracy. If *A *= 0, the result is a poor approximation, as the most sparse solution is a zero weight vector, **w **= **0**. When *A *= ∞, deviations are not allowed and a non-sparse **w **is found if the problem is feasible.

#### LP formulation: general class of linear functions

Consider the robust regression function *f*(.; **w**). For the general class of linear functions, *f*(**x**; **w**) = **w**^⊤^**x**, an element of the parameter vector can be zero, *w*_*j *_= 0, or non-zero, *w*_*j *_≠ 0. When *w*_*j *_> 0, the predictor variable *j *makes a positive contribution to the linear interaction model, whereas if *w*_*j *_< 0, the contribution is negative. Since the representation of a genetic network considered here is an undirected graph and thus the connectivity matrix is symmetric, the interactions (edges) in a SLGN are not categorized as activation or inhibition.

For the general class of linear functions *f*(**x**; **w**) = **w**^⊤^**x**, an element of the weight vector **w **should be non-zero, *w*_*j *_≠ 0. Then, the LASSO problem

(3) $$\begin{array}{ll} \underset{w,v}{\text{minimize}} & \sum_{i=1}^{I}\left|v_{i}\right|\\ \text{subject to} & w^{\text{T}}x_{i}+v_{i}=y_{i}\\ & {\left\|w\right\|}_{1}\leq A. \end{array}$$

can be posed as the following LP

(4) $$\begin{array}{ll} \underset{u,v,\xi ,\xi *}{\text{minimize}} & \sum_{i=1}^{I}\left(\xi_{i}+\xi_{i}^{*}\right)\\ \text{subject to} & {\left(u-v\right)}^{\text{T}}x_{i}+\xi_{i}-\xi_{i}^{*}=y_{i}\\ & {\left(u+v\right)}^{\text{T}}1\leq A\\ & u\geq 0;v\geq 0\\ & \xi_{i}\geq 0;\xi_{i}^{*}\geq 0 \end{array}$$

by substituting **w **= **u **- **v**, ||**w**||_1 _= (**u **+ **v**)^⊤^**1**, |*v*_*i*_| = *ξ*_*i *_+ $\xi_{i}^{*}$ and *v*_*i *_= *ξ*_*i *_- $\xi_{i}^{*}$. The user-defined parameter *A *controls the upper bound of the *l*_1 _norm of the weight vector and thus the trade-off between sparsity and accuracy. Problem (4) is an LP in (2*N *+ 2*I*) variables, *I *equality constraints, **1 **inequality constraints and (2*N *+ 2*I*) non-negativity constraints.

#### LP formulation: positive class of linear functions

An optimization problem with fewer variables than problem (4) can be formulated by considering a weaker class of linear functions. For the positive class of linear functions *f*(**x**; **w**) = **w**^⊤^**x**, an element of the weight vector **w **should be non-negative, *w*_*j *_≥ 0. Then, the LASSO problem (Equation 3) can be posed as the following LP,

(5) $$\begin{array}{ll} \underset{w,\xi ,\xi *}{\text{minimize}} & \sum_{i=1}^{I}\left(\xi_{i}+\xi_{i}^{*}\right)\\ \text{subject to} & w^{\text{T}}x_{i}+\xi_{i}-\xi_{i}^{*}=y_{i}\\ & w^{\text{T}}1\leq A\\ & w\geq 0\\ & \xi_{i}\geq 0;\xi_{i}^{*}\geq 0. \end{array}$$

Problem (5) is an LP with (*N *+ 2*I*) variables, *I *equality constraints, **1 **inequality constraints, and (2*N *+ 2*I*) non-negativity constraints.

In most transcript profiling studies, the number of genes monitored is considerably greater than the number of profiles produced, *N *≫ *I*. Thus, an LP based on a restrictive positive linear class of functions and involving (*N *+ 2*I*) variables (Problem (5)) offers substantial computational advantages over a formulation based on a general linear class of functions and involving (2*N *+ 2*I*) variables (Problem (4)). LPs involving thousands of variables can be solved efficiently using extant software and tools.

To estimate a graph G, the training points for the *n*^*th *^gene, $D_{n}$, are used to solve a sparse linear regression problem posed as a LASSO and formulated as an LP. The outcome of such regression analysis is a sparse weight vector **w**_*n *_whose small number of non-zero elements specify which genes influence gene *n*. Aggregating the *N *sparse weight vectors produced by solving *N *independent sparse linear regression problems [**w**_1_,..., **w**_*N*_], yields the matrix **W **that parameterizes the graph.

### Statistical assessment of LP-SLGNs: Error, Sparsity and Leave-One-Out (LOO) Error

The "Sparsity" of a graph G is the average degree of a node

(6) $$\text{Sparsity}=\frac{1}{N}\sum_{n=1}^{N}k_{n}=\frac{1}{N}\sum_{n=1}^{N}{\left\|w_{n}\right\|}_{0}$$

where ||**w**_*n*_||_0 _is the *l*_0 _norm of the weight vector for node *n*.

Unfortunately, the small number of available training points (*I*) means that the empirical error will be optimistic and biased. Consequently, the Leave-One-Out (LOO) Error is used to analyze the stability and generalization performance of the method proposed here.

Given a training set $D_{n}$ = [(**x**_*n*1_, *y*_*n*1_),..., (**x**_*nI*_, *y*_*nI*_)], two modified training sets are built as follows

• Remove the *i*th element: $D_{n}^{\backslash i}=D_{n}\backslash \{(x_{ni},y_{ni})\}$

• Change the *i*th element: $D_{n}^{i}=D_{n}\backslash \{(x_{ni},y_{ni})\}\cup (x^{\prime },y^{\prime })$, where (**x**', *y*') is any point other than one in the training set $D_{n}$

The Leave-One-Out Error of a graph G, LOO Error, is the average over the *N *nodes of the LOO error of every node. The LOO error of node *n*, *LOO*_*error*_($D_{n}$), is the average over the *I *training points of the magnitude of the discrepancy between the actual response, *y*_*ni*_, and the predicted linear response, $f^{\backslash i}(x_{ni};w_{n}^{\backslash i})=w_{n}^{\backslash i\text{T}}x_{ni}$,

(7) $$\begin{array}{l} \text{LOO Error}=\frac{1}{N}\sum_{n=1}^{N}LOO_{error}(D_{n})\\ LOO_{error}(D_{n})=\frac{1}{I}\sum_{n=1}^{I}\left|y_{ni}-f^{\backslash i}(x_{ni};w_{n}^{\backslash i})\right| \end{array}$$

The parameter $w_{n}^{\backslash i}$ of the function $f^{\backslash i}(x_{ni};w_{n}^{\backslash i})$ is learned using the modified training set $D_{n}^{\backslash i}$.

#### A bound for the Generalization Error of a graph

A key issue in the design of any machine learning system is an algorithm that has low generalization error.

Here, the Leave-One-Out (LOO) error is utilized to estimate the accuracy of the LP-based algorithm employed to learn the structure of a SLGN. In this section, a bound on the generalization error based on the LOO Error is derived. Furthermore, a low "LOO Error" of the method proposed here is shown to signify good generalization.

The generalization error of a graph G, Error, is the average over all *N *nodes of the generalization error of every node, *Error*($D_{n}$),

(8) $$\begin{array}{lll} \text{Error} & = & \frac{1}{N}\sum_{n=1}^{N}Error(D_{n})\\ Error(D_{n}) & = & E_{D_{n}}[l(f;x,y)]\\ l(f;x,y) & = & \left|y-w_{n}^{\text{T}}x\right| \end{array}$$

The parameter **w**_*n *_is learned from $D_{n}$ as follows,

(9) $$w_{n}=\underset{||w|{|}_{1}\leq t}{\arg\min}\frac{1}{I}\sum_{i=1}^{I}l(w,(x_{ni},y_{ni}))$$

The approch is based on the following Theorem (for details, see [43]),

**Theorem 1**. *Given a training set S *= {**z**_1_,..., **z**_*m*_} *of size m, let the modified training set be S*^*i *^= {**z**_1_,..., **z**_*i*-1_, ${z^{\prime }}_{i}$, **z**_*i*+1_,..., **z**_*m*_}, *where the i^th ^element *${z^{\prime }}_{i}$*has been changed and is drawn from the data space Z but independent of S. Let F *= *Z*^*m *^→ R*be any measurable function for which there exists constants c*_*i *_(*i *= 1,..., *m*) such that

$$\begin{array}{c} \underset{S\varepsilon Z^{m},{z^{\prime }}_{i}\varepsilon Z}{sup}|(F(S)-(F(S^{i})|\leq c_{i},\\ then\;P_{s}[(F(S)-E_{s}[F(S)])\geq \varepsilon ]\leq e^{-2\varepsilon^{2}}/\sum_{i=1}^{m}c_{i}^{2}. \end{array}$$

Elsewhere [44], the above was given as Theorem 2.

**Theorem 2**. *Consider a graph *G*with N nodes. Let the data points for the n^th ^node be *$D=\{(x_{ni},y_{ni})|;x_{ni}\in R^{N};y_{ni}\in R;i=1,...,I\}$*where *(**x**_*ni*_, *y*_*ni*_) *are iid. Assume that *||**x**_*ni*_||_∞ _≤ *d and *|*y*_*ni*_| ≤ *b. Let *$f:R^{N}\to R$*and y *= *f*(**x**; **w**) = **w**^⊤^**x**. *Using techniques from *[44], *it can be stated that for *0 ≤ *δ *≤ 1 *and with probability at least *1 - *δ over a random draw of the sample graph *G,

(10) $$Error\leq LOO\;Error+2td+\left(6td+\frac{b}{1}\right)\sqrt{\frac{I\ln\left(\frac{1}{\delta }\right)}{2}}$$

*where t is the l*_1 _*norm of the weight vector *||**w**||_1_. *LOO Error and Error are calculated using Equation 7 and Equation 8 respectively*.

PROOF. "Random draw" means that if the algorithm is run for different graphs, one graph from the set of learned graphs is selected at random. The proposed bound of generalization error will be true for this graph with high probability. This term is unrelated to term "Random graph" used in Graph Theory.

The following proof makes use of Holder's Inequality.

(11) $$\begin{array}{ll} & {\left\|\left|y_{ni}-f(x_{ni};w_{n})|-|y_{ni}-f^{\backslash i}(x_{ni};w_{n}^{\backslash i})\right|\right\|}_{\infty }\\ \leq & \left|w_{n}^{\text{T}}x_{ni}-w_{n}^{\backslash i\text{T}}x_{ni}\right|\\ \leq & {\left\|\left(w_{n}-w_{n}^{\backslash i}\right)\right\|}_{1}{\left\|x_{ni}\right\|}_{\infty }\\ \leq & 2{\left\|w_{n}\right\|}_{1}d\\ \leq & 2td. \end{array}$$

A bound on the Empirical Error can be found as

(12) $$\begin{array}{lll} \max(|y_{ni}-f(x_{ni};w_{n})|) & \leq & \left|y_{ni}|+|w_{n}^{\text{T}}x_{ni}\right|\\ & \leq & b+{\left\|w_{n}\right\|}_{1}{\left\|x_{ni}\right\|}_{\infty }\\ & \leq & b+td. \end{array}$$

Let *Error*($D_{n}^{\backslash i}$) be the Generalization Error after training with $D_{n}^{\backslash i}$. Then using Equation 11

(13) $$\begin{array}{ll} & \left|Error(D_{n})-Error(D_{n}^{\backslash i})\right|\\ = & \left|E_{D_{n}}[|y-f(x;w_{n})|]-E_{D_{n}}[|y-f^{\backslash i}(x;w_{n}^{\backslash i})|]\right|\\ \leq & {\left\|\left|y_{ni}-f(x_{ni};w_{n})|-|y_{ni}-f^{\backslash i}(x_{ni};w_{n}^{\backslash i})\right|\right\|}_{\infty }\\ \leq & 2td. \end{array}$$

Let *Error*($D_{n}^{i}$) be the Generalization Error after training with $D_{n}^{i}$. Then using Equation 13

(14) $$\begin{array}{ll} & \left|Error(D_{n})-Error(D_{n}^{i})\right|\\ = & \left|\left(Error(D_{n})-Error(D_{n}^{\backslash i}))-(Error(D_{n}^{\backslash i})-Error(D_{n}^{i})\right)\right|\\ \leq & \left|Error(D_{n})-Error(D_{n}^{\backslash i})|+|Error(D_{n}^{\backslash i})-Error(D_{n}^{i})\right|\\ \leq & 4td. \end{array}$$

If *LOO*_*error*_($D_{n}^{i}$) is the LOO error when the training set is $D_{n}^{i}$, then using Equation 11 and Equation 12,

(15) $$\begin{array}{ll} & \left|LOO_{error}(D_{n})-LOO_{error}(D_{n}^{i})\right|\\ = & \frac{1}{I}|\sum_{j\neq i}\left(|y_{ni}-f^{\backslash j}(x_{nj};w_{n}^{\backslash j})|-|y_{ni}-f^{i\backslash j}(x_{nj};w_{n}^{i\backslash j})|\right)\\ & +(|y_{ni}-f^{\backslash i}(x_{nj};w_{n}^{\backslash i})|-|{y^{\prime }}_{ni}-f^{\backslash i}({x^{\prime }}_{ni};w_{n}^{\backslash i})|)|\\ \leq & \frac{1}{I}|\sum_{j\neq i}\left|f^{\backslash j}(x_{nj};w_{n}^{\backslash j})-f^{i\backslash j}(x_{nj};w_{n}^{i\backslash j})\right|\\ & +(|y_{ni}-f^{\backslash i}(x_{ni};w_{n}^{\backslash i})|-|{y^{\prime }}_{ni}-f^{\backslash i}({x^{\prime }}_{ni};w_{n}^{\backslash i})|)|\\ \leq & \frac{1}{I}|\sum_{j\neq i}\left|{\left(w_{n}^{\backslash j}-w_{n}^{i\backslash j}\right)}^{\text{T}}x_{j}\right|+(b+td)|\\ \leq & \frac{1}{I}|(I-1)2td|(b+td)|\\ \leq & 2td+\frac{b}{I}. \end{array}$$

Thus, the random variable (Error - LOO Error) satisfies the condition of Theorem 1. Using Equation 14 and Equation 15, the condition is

(16) $$\begin{array}{ll} & \underset{G,(x,y)}{\sup}|(\text{Error}-\text{LOO Error})-({\text{Error}}^{i}-{\text{LOO Error}}^{i})|\\ \leq & \left|\text{Error}-{\text{Error}}^{i}|+|\text{LOO Error}-{\text{LOO Error}}^{i}\right|\\ = & \frac{1}{N}\sum_{n=1}^{N}\left(|Error(D_{n})-Error(D_{n}^{i})\right|\\ & +|LOO_{error}^{i}(D_{n})-LOO_{error}(D_{n}^{i})|)\\ \leq & \frac{1}{N}\sum_{n=1}^{N}\left(6td+\frac{b}{I}\right)\\ = & 6td+\frac{b}{I}. \end{array}$$

Where Error^*i *^is the Generalization of graph G and LOO Error^*i *^is LOO Error of graph G when the *i*^*th *^data points for all genes are changed. Thus, only a bound on the expectation of the random variable (Error - LOO Error) is needed. Using Equation 11,

$$\begin{array}{ll} & \text{E}[\text{Error}-\text{LOO Error}]\\ = & \frac{1}{N}\sum_{n=1}^{N}(\frac{1}{I}\sum_{i=1}^{n}\left(|y_{ni}-f(x_{ni};w_{n})|-|y_{ni}-f^{\backslash i}(x_{ni};w_{n}^{\backslash i})|)\right)\\ \leq & 2td. \end{array}$$

Hence, Theorem 1 can be used to state that if Equation 16 holds, then

(17) $$\begin{array}{l} \text{P}[((\text{Error}-\text{LOO Error})]-E[\text{Error}-\text{LOO Error}])\geq \varepsilon ]\\ \leq \exp\left(\frac{-2\varepsilon^{2}}{I{\left(6td+\frac{b}{I}\right)}^{2}}\right). \end{array}$$

By equating the right hand side of Equation 17 to *δ*

$$\text{P}[\text{Error}<\text{LOO Error}+2td+\left(6td+\frac{b}{I}\right)\sqrt{\frac{I\;ln\left(\frac{1}{\delta }\right)}{2}}]\geq (1-\delta ).$$

Given this bound on the generalization error, a low LOO Error in the method proposed here signifies good generalization.   □

### Implementation and numerical issues

Prototype software implementing the two LP-based formulations of sparse regression was written using the tools and solvers present in the commercial software MATLAB [45]. Software is available in "Additional file 1" named as "LP-SLGN.tar". It should be straightforward to develop an implementation using C and R wrapper functions for lpsolve [46], a freely available solver for linear, integer and mixed integer programs. The outcome of regression analysis is an optimal weight vector **w**. Limitations in the numerical precision of solvers means that an element is never exactly zero but a small finite number. Once a solver finds a vector **w**, a "small" user-defined threshold is used to assign zero and non-zero elements. If the value produced by a solver is greater than the threshold *w*_*j *_= 1, otherwise *w*_*j *_= 0. Here, a cut-off of 10^-8 ^was used.

The computational experiments described here were performed on a large shared machine. The hardware specifications are 6 × COMPAQ AlphaServers ES40 with 4 CPUs per server with 667 MHz, 64 KB + 64 KB primary cache per CPU, 8 MB secondary cache per CPU, 8 GB memory with 4 way interleaving, 4 * 36 GB 10 K rpm Ultra3 SCSI disk drive, and 2*10/100 Mbit PCI Ethernet Adapter. However, the programs can be run readily on a powerful PC. For the MATLAB implementation of the LP formulation based on the general class of linear functions, the LP took a few seconds of wall clock time. An additional few seconds were required to read in files and to set up the problem.

## Results and discussion

### DREAM2 In-Silico-Network Challenges data

#### Statistical assessment of LP-SLGNs estimated from simulated data

**Figure 1:**
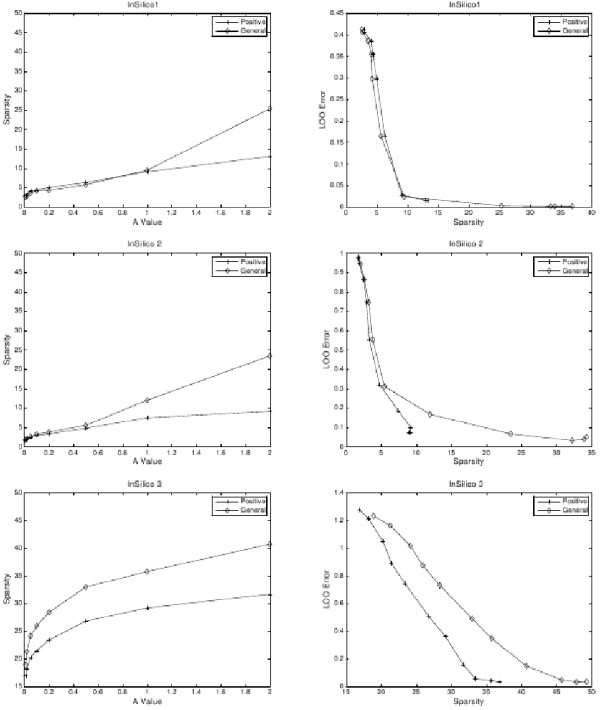
**Quantitative evaluation of the INSILICO network models**. Statistical assessment of the LP-SLGNs estimated from the INSILICO1, INSILICO2, and INSILICO3 DREAM2 data sets [36]. The left column shows plots of "Sparsity" (Equation 6) versus the user-defined parameter A (Equation 3). The right column shows plots of "LOO Error" (Equation 7) versus Sparsity. Each plot shows results for an LP formulation based on a general class of linear functions (diamond) and a positive class of linear functions (cross).

LP-SLGNs were estimated from the INSILICO1, INSILICO2, and INSILICO3 data sets using both LP formulations and different settings of the user-defined parameter *A *which controls the upper bound of the *l*_1 _norm of the weight vector and hence the trade-off between sparsity and accuracy. The results are shown in Figure 1. For all data sets, smaller values of *A *yield sparser graphs (left column) but Sparsity comes at the expense of higher LOO Error (right column). Higher *A *values produce graphs where the average degree of a node is larger (left column). The LOO Error decreases with increasing Sparsity (right column). The maximum Sparsity occurs at high *A *values and is equal to the number of genes *N*.

LP-SLGNs based on the general class of linear functions were estimated using the parameter *A *= 1. For the INSILICO1 data set, the Sparsity is ~10. For the INSILICO2 data set, the Sparsity is ~13. For the INSILICO3 data set, the Sparsity is ~35.

The learned LP-SLGNs were evaluated using a script provided by the DREAM2 Project [38]. The results are shown in Table 1. The INSILICO2 LP-SLGN is considerably better than the network predicted by Team80, Which team is the top-ranked team in the DREAM2 competition (Challenge 4). The INSILICO1 LP-SLGN is comparable to the predicted network of Team70, the top ranked team, but better than that of Team 80, the second-ranked team. Team rankings are not available for the INSILICO3 dataset. The predicted networks by LP-SLGN can be found in "Additional file 2" named as "Result.tar".

**Table 1:** Comparison of the networks – undirected graphs – produced by three different approaches: the LP-based method proposed here, and techniques proposed by the top two teams of the DREAM2 competition (Challenge 4).

Dataset	Team	Precision at *k*^*th *^correct prediction				Area Under PR Curve	Area Under ROC Curve
		k = 1	k = 2	k = 5	k = 20		
INSILICO1	Team 70	1.000000	1.000000	1.000000	1.000000	0.596721	0.829266
	Team 80	0.142857	0.181818	0.045045	0.059524	0.070330	0.459704
	LP-SLGN	0.083333	0.086957	0.089286	0.117647	0.087302	0.509624

INSILICO2	Team 80	0.333333	0.074074	0.102041	0.069204	0.080266	0.536187
	Team 70	0.142857	0.250000	0.121320	0.081528	0.084303	0.511436
	LP-SLGN	1.000000	1.000000	0.192308	0.183486	0.200265	0.750921

INSILICO3	LP-SLGN	0.068966	0.068966	0.068966	0.068966	0.068966	0.500000

For the first *k *predictions (ranked by score, and for predictions with the same score, taken in the order they were submitted in the prediction files), the DREAM2 evaluation script defines precision as the fraction of correct predictions of *k*, and recall as the proportion of correct predictions out of all the possible true connections. The other metrics are the Precision-Recall (PR) and Receiver Operating Characteristics (ROC) curves.

### S. cerevisae transcript profiling data

#### Statistical assessment of LP-SLGNs estimated from real data

**Figure 2:**
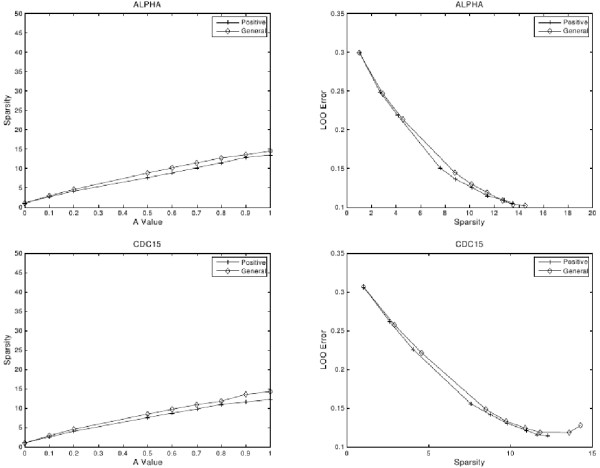
**Quantitative evaluation of the S. cerevisiae network models**. Statistical assessment of the LP-SLGNs estimated from the *S. cerevisiae *ALPHA and CDC15 data sets [37]. The left column shows plots of "Sparsity" (Equation 6) versus the user-defined parameter *A *(Equation 3). The right column shows plots of "LOO Error" (Equation 7) versus Sparsity. Each plot shows results for an LP formulation based on a general class of linear functions (diamond) and a positive class of linear functions (cross).

LP-SLGNs for the ALPHA and CDC15 data sets were estimated using both LP formulations and different settings of the user-defined parameter *A*. The learned undirected graphs were evaluated by computing LOO Error (Equation 7), a quantity indicating generalization performance, and Sparsity (Equation 6), a quantity based on the degree of each node. The results are shown in Figure 2. LP formulations based on a weaker positive class of linear functions (cross) and a general class of functions linear (diamond) produce similar results. However, the formulation based on a positive class of linear functions can be solved more quickly because it has fewer variables. For both data sets, smaller *A *values yield sparser graphs (left column) but sparsity comes at the expense of higher LOO Error (right column). For high *A *values, the average degree of a node is larger (left column). The LOO Error decreases with the increase of Sparsity (right column). The maximum Sparsity occurs at high *A *values and is equal to the number of genes *N*. The minimum LOO Error occurs at *A *= 1 for ALPHA and *A *= 0.9 for CDC15; the Sparsity is ~15 for these *A *values. The degree of most of the nodes in the LP-SLGNs lies in the range 5–20, *i.e*., most of the genes are influenced by 5–20 other genes.

**Figure 3:**
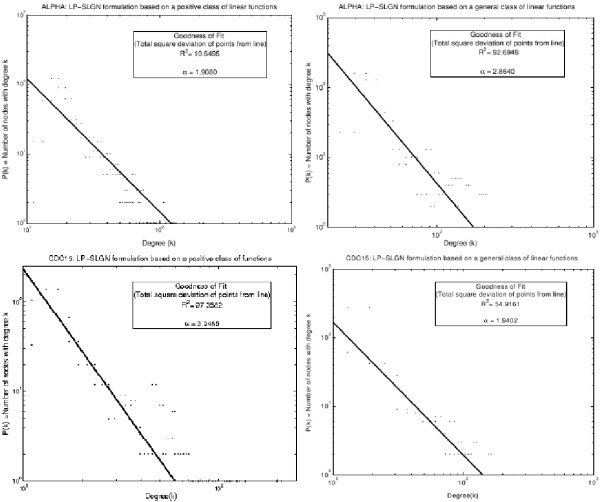
**Node degree distribution of the S. cerevisiae network models**. The distribution of the degrees of nodes in the LP-SLGNs estimated from the *S. cerevisiae *ALPHA and CDC15 data sets using both LP formulations (a general class of linear functions; a positive class of linear functions). The best fit straight line in each logarithmic plot means that the number *P*(*k*) of nodes with degree *k *follows a power law, *P*(*k*) ∝ *k*^-*α*^. The goodness of fit and the value of the exponent *α *are given.

Figure 3 shows logarithmic plots of the distribution of node degree for the ALPHA and CDC15 LP-SLGNs. In each case, the degree distribution roughly follows a straight line, *i.e*., the number of nodes with degree *k *follows a power law, *P*(*k*) = *βk*^-*α *^where *β*, *α *∈ **R**. Such a power-law distribution is observed in a number of real-world networks [47]. Thus, the connectivity pattern of edges in LP-SLGNs are consistent with known biological networks.

#### Biological evaluation of S. cerevisiae LP-SLGNs

**Figure 4:**
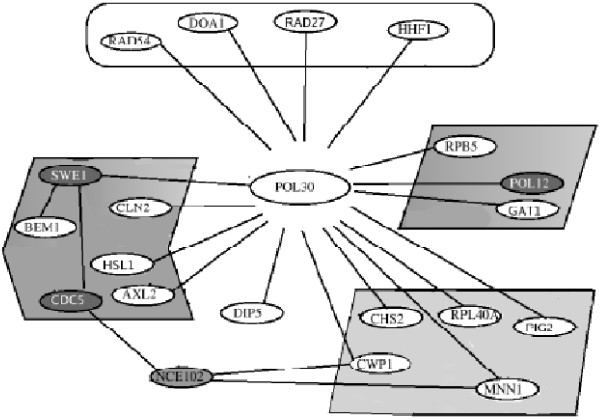
**The local environment of POL30 in the S. cerevisiae network models**. Genes connected to POL30 in the LP-SLGNs estimated from the *S. cerevisiae *ALPHA and CDC15 data sets (further information about the proteins encoded by the genes shown can found in Additional File 1). Genes in black (SWE1, POL12, CDC5, NCE102) were assigned to the same expression cluster in the original transcript profiling study [37]. Functionally related genes are boxed.

The profiling data examined here were the outcome of a study of the cell cycle in *S. cerevisiae *[37]. The published study described gene expression clusters (groups of genes) with similar patterns of abundance across different conditions. Whereas two genes in the same expression cluster have similarly shaped expression profiles, two genes linked by an edge in an LP-SLGN model have linearly related abundance levels (a non-zero element in the connectivity matrix of the undirected graph, *w*_*ij *_≠ 0). The ALPHA and CDC15 LP-SLGNs were evaluated from a biological perspective by manual analysis and visual inspection of LP-SLGNs estimated using the LP formulation based on a general class of linear functions and *A *= 1.0^1^. Figure 4 shows a small, illustrative portion of the ALPHA and CDC15 LP-SLGNs centered on the POL30 gene. For each the genes depicted in the figure, the *Saccharomyces *Genome Database (SGD) [48] description, Gene Ontology (GO) [49] terms and InterPro [50] protein domains (when available) are listed in "Additional file 3" named as "Supplementary.pdf". The genes connected to POL30 encode proteins that are associated with maintenance of genomic integrity (DNA recombination repair, RAD54, DOA1, HHF1, RAD27), cell cycle regulation, MAPK signalling and morphogenesis (BEM1, SWE1, CLN2, HSL1, ALX2/SRO4), nucleic acid and amino acid metabolism (RPB5, POL12, GAT1), and carbohydrate metabolism and cell wall biogenesis (CWP1, RPL40A, CHS2, MNN1, PIG2). Physiologically, the KEGG [51] pathways associated with these genes include "Cell cycle" (CDC5, CLN2, SWE1, HSL1), "MAPK signaling pathway" (BEM1), "DNA polymerase" (POL12), "RNA polymerase" (RPB5), "Aminosugars metabolism" (CHS2), "Starch and sucrose metabolism" (RAD54), "High-mannose type N-glycan biosynthesis" (MNN1), "Purine metabolism" (POL12, RPB5), "Pyrimidine metabolism" (POL12, RPB5), and "Folate biosynthesis" (RAD54).

The learned LP-SLGNs provide a forum for generating biological hypotheses and thus directions for future experimental investigations. The edge between SWE1 and BEM1 indicates that the transcript levels of these two genes exhibit a linear relationship; the physical interactions section of their SGD [48] entries indicates that the encoded proteins interact. These results suggests that cellular and/or environmental factor(s) that perturb the transcript levels of both SWE1 and BEM1 may affect cell polarity and cell cycle. NCE102 is connected to genes involved in cell cycle regulation (CDC5) and cell wall remodelling (CWP1, MNN1). A recent report indicates that the transcript level of NCE102 changes when *S. cerevisiae *cells expressing human cytochrome CYP1A2 are treated with the hepatotoxin and hepatocarcinogen aflatoxin B1 [52]. Thus, this uncharacterized gene may be part of a cell cycle-related response to genotoxic and/or other stress.

Studies of the yeast NCE102 gene may be relevant to human health and disease. The protein encoded by NCE102 was used as the query for a PSI-BLAST [53] search using the WWW interface to the software at NCBI and default parameter settings. Amongst the proteins exhibiting statistically significant similarity (E-value ≪ 1e - 05) were members of the mammalian physin and gyrin families, four-transmembrane domain proteins with roles in vesicle trafficking and membrane morphogenesis [54]. Human synaptogyrin 1 (SYNGR1; E-value ~ 1e - 28) has been linked to schizophrenia and bipolar disorder [55].

## Conclusion

Like this work, a previous study [17] framed the question of deducing the structure of a genetic network from transcript profiling data as a problem of sparse linear regression. The earlier investigation utilized SVD and robust regression to deduce the structure of a network. In particular, the set of all possible networks was characterized by a connectivity matrix **A **defined by the equation **A **= **A**_0 _+ **CV**^⊤^. The matrix **A**_0 _computed from the data matrix **E **via SVD can be seen as the best, in the *l*_2 _norm sense, connectivity matrix which can generate the data. The matrix **V **is the right singular vectors of **E**. The requirement of a sparse graph was enforced by choosing the matrix **C **such that most of the entries in the matrix **A **are zero. An approximate solution to the original equation was obtained by posing it as a robust regression problem such that **CV**^⊤ ^= -**A**_0 _was enforced approximately. This new regression problem was solved by formulating an LP that included an *l*_1 _norm penalty for deviations from equality. In contrast, the solution to the sparse linear regression problem proposed here avoids the need for SVD by formulating the problem directly within the framework of LOO Error and Empirical Risk Minimization and enforcing sparsity via an upper bound on the *l*_1 _norm of the weight vector, *i.e*., the original regression problem is posed as a series of LPs. The virtues of this LP-based approach for learning the structure of SLGNs include (i) the method is tractable, (ii) a sparse graph is produced because very few predictor variables are used, (iii) the network model can be parametrized by a positive class of linear functions to produce LPs with few variables, (iv) efficient algorithms and resources for solving LPs in many thousands of variables and constraints are widely and freely available, and (v) the learned network models are biologically reasonable and can be used to devise hypotheses for subsequent experimental investigation.

Another method for deducing the structure of genetic networks framed the task as one of finding a sparse inverse covariance matrix from a sample covariance matrix [56]. This approach involved solving a maximum likelihood problem with an *l*_1_-norm penalty term added to encourage sparsity in the inverse covariance matrix. The algorithms proposed for this can do no better than *O*(*N*^3^). Better results were achieved by incorporating prior information about error in the sample covariance matrix. In contrast, the LP-based approach to the sparse linear regression problem avoids calculation of a covariance matrix and does not require prior knowledge. Furthermore, the approach proposed here can learn networks with thousands genes in a few minutes on a personal computer.

The quality and utility of the learned LP-SLGNs could be enhanced in a number of ways. The network models examined here were estimated from transcript profiles that were subject to minimal data pre-processing. Appropriate low-level analysis of profiling data is known to be important [57] so estimating network models from suitably processed data would improve both their accuracy and reliability. The biological predictions were made by visual inspection of a small portion of the LP-SLGNs and in an *ad-hoc *manner. Hypotheses could be generated in a systematic manner by exploiting statistical and topological properties of sparse undirected graphs. For example, a feature that unites the local and global aspects of a node is its "betweenness", the influence the node has over the spread of information through the graph. The random-walk betweenness centrality of a node [58] captures the proportion of times a node lies on the path between other nodes in the graph. Nodes with high betweenness but small degree (low connectivity) are likely to play a role in maintaining the integrity of the graph. Betweenness values could be computed from a weighted undirected graph created from an ensemble of LP-SLGNs produced by varying the user-defined parameter *A*. Given a variety of LP-SLGNs estimated from data, the cost of an edge could be equated with the frequency with it appears in the learned network models. For the profiling data analyzed here, genes with high betweenness and low degree may have important but unrecognized roles in the *S. cerevisae *cell cycle and hence correspond to good candidates for experimental investigations of this phenomenon.

The weighted sparse undirected graph described above could serve as the starting point for integrated computational – experimental studies aimed at learning the topology and probability parameters of a probabilistic directed graphical model, a more realistic representation of a genetic network because the edges are oriented and the statistical framework provides powerful tools for asking questions related to the values of variables (nodes) given the values of other variables (inference), handling hidden or unobserved variables, and so on. However, estimating the topology of probabilistic directed graphical model representations of genetic networks from transcript profiling data is challenging [59]. Genes with high betweenness and low degree could be targeted for intervention studies whereby a specific gene would be knocked out in order to determine the orientation of edges associated with it (see, for example, [60]). A variety of theoretical improvements are possible. An explicit model for uncertainty in transcript profiling data could be used to formulate and then solve robust sparse linear regression problems and hence produce models of genetic networks that are more resilient to variation in training data than those generated using the Huber loss function considered here. Expanding the class of interactions from linear models to non-linear models is an important research topic.

## Competing interests

The authors declare that they have no competing interests.

## Authors' contributions

SB, CB and ISM conceived and developed the computational ideas presented in this work. SB and CB formulated the optimization problems, wrote the software and performed the experiments. NC analyzed the data with contributions from the other authors. All authors read and approved the final version of the manuscript.

## Note

^1^

## Supplementary Material

Additional file 1 **The codes of LP-SLGN are available here.** Click here for file

Additional file 2 **Predicted networks obtained for InSilico and Yeast dataset using LP-SLGN are available here.** Click here for file

Additional file 3 **Information about the proteins encoded by the genes depicted in Figure **4. For each gene, the *Saccharomyces *Genome Database (SGD) [48] description, Gene Ontology (GO) [49] terms and InterPro [50] protein domains are listed (when available). Click here for file
