# Supplementary material for: A linear programming approach for estimating the structure of a sparse linear genetic network from transcript profiling data
Source: Algorithms Mol Biol. 2009 Feb 24;4:5. doi: 10.1186/1748-7188-4-5 (PMC2654898; doi:10.1186/1748-7188-4-5)
Supplement: Additional file 3 — Information about the proteins encoded by the genes depicted in Figure 4. For each gene, the Saccharomyces Genome Database (SGD) [48] description, Gene Ontology (GO) [49] terms and InterPro [50] protein domains are listed (when available). [file 1748-7188-4-5-S3.pdf]

Appendix I

Table 1: Information about the proteins encoded by the genes depicted in Figure 3. For each gene, the *Saccharomyces* Genome Database (SGD) description, Gene Ontology (GO) terms and InterPro protein domains are listed (when available).

Name	Description
RAD54 (YGL163C)	**SGD** DNA-dependent ATPase, stimulates strand exchange by modifying the topology of double-stranded DNA; involved in the recombinational repair of double-strand breaks in DNA during vegetative growth and meiosis; member of the SWI/SNF family. **GO** DNA topoisomerase activity, DNA-dependent ATPase activity, ATP binding, DNA binding, helicase activity, hydrolase activity, nucleic acid binding, nucleotide binding. chromatin remodeling, heteroduplex formation, telomerase-independent telomere maintenance, double-strand break repair via synthesis-dependent strand annealing, DNA repair, response to DNA damage stimulus. Nucleus. **Interpro** DEAD/DEAH box helicase, N-terminal; The DEAD box helicases are involved in various aspects of RNA metabolism, including nuclear transcription, pre-mRNA splicing, ribosome biogenesis, nucleocytoplasmic transport, translation, RNA decay and organellar gene expression. Helicase, C-terminal; SNF2-related; SNF2 functions as the ATPase component of the SNF2/SWI multisubunit complex, which utilises energy derived from ATP hydrolysis to disrupt histone-DNA interactions, resulting in the increased accessibility of DNA to transcription factors.
DOA1 (YKL213C)	**SGD** WD repeat protein required for ubiquitin-mediated protein degradation, forms complex with Cdc48p, plays a role in controlling cellular ubiquitin concentration; also promotes efficient NHEJ in postdiauxic/stationary phase. **GO** double-strand break repair via nonhomologous end-joining; ubiquitin-dependent protein catabolism. cytoplasm; nucleus. **Interpro** WD-40 repeats (also known as WD or beta-transducin repeats) are short $\sim$ 40 amino acid motifs, often terminating in a Trp-Asp (W-D) dipeptide. The underlying common function of all WD-repeat proteins is coordinating multi-protein complex assemblies, where the repeating units serve as a rigid scaffold for protein interactions.
RAD27 (Ykl113cp)	**SGD** 5' to 3' exonuclease, 5' flap endonuclease, required for Okazaki fragment processing and maturation as well as for long-patch base-excision repair; member of the S. pombe RAD2/FEN1 family. **GO** Nucleus. protein binding; 5'-flap endonuclase activity. DNA repair; DNA replication; DNA synthesis during DNA repair; double-strand break repair via non homologous end joining; replicative cell aging; telomere maintanace. **Interpro** The helix-hairpin-helix (HhH) motif is an around 20 amino acids domain present in prokaryotic and eukaryotic non-sequence-specific DNA binding proteins. These HhH motifs are observed in DNA repair enzymes and in DNA polymerases.
HHF1 (YBR009C)	**SGD** One of two identical histone H4 proteins (see also HHF2); core histone required for chromatin assembly and chromosome function; contributes to telomeric silencing; N-terminal domain involved in maintaining genomic integrity. **GO** DNA binding. chromatin assembly or disassembly. nuclear nucleosome. **Interpro** Histone-fold/TFIID-TAF/NF-Y; Histone core; Histone H4.
RPB5 (YBR154C)	**SGD** RNA polymerase subunit ABC27, common to RNA polymerases I, II, and III; contacts DNA and affects transactivation. **GO** DNA-directed RNA polymerase activity. transcription from RNA polymerase I promoter; transcription from RNA polymerase II promoter; transcription from RNA polymerase III promoter. DNA-directed RNA polymerase I complex; DNA-directed RNA polymerase II, core complex; DNA-directed RNA polymerase III complex. **Interpro** RNA polymerase Rpb5, N-terminal; RNA polymerase subunit, RPB5.
POL12 (YBL035C)	**SGD** B subunit of DNA polymerase α-primase complex, required for initiation of DNA replication during mitotic and premeiotic DNA synthesis; also functions in telomere capping and length regulation. **GO** α DNA polymerase activity. DNA replication initiation; DNA replication, synthesis of RNA primer; lagging strand elongation. α DNA polymerase:primase complex; nuclear membrane; nucleus. **Interpro** DNA polymerase α subunit B.

Name	Description
GAT1 (Yfl021wp)	**SGD** Transcriptional activator of genes involved in nitrogen catabolite repression, member of the GATA family of DNA binding proteins; activity and localization regulated by nitrogen limitation and Ure2p **GO** cytosol; nucleus. specific RNA polymerase II transcription factor activity; transcriptional activator activity. positive regulation of transcription; regulation of nitrogen utilization; transcription initiation from RNA polymeras. **Interpro** A number of transcription factors (including erythroid-specific transcription factor and nitrogen regulatory proteins), specifically bind the DNA sequence (A/T)GATA(A/G) in the regulatory regions of genes.
RPL40A (YIL148W)	**SGD** Fusion protein, identical to Rpl40Bp, that is cleaved to yield ubiquitin and a ribosomal protein of the large (60S) ribosomal subunit with similarity to rat L40; ubiquitin may facilitate assembly of the ribosomal protein into ribosomes. **GO** protein tag; structural constituent of ribosome. protein biosynthesis; protein ubiquitination; ribosome biogenesis and assembly. cytosolic large ribosomal subunit (sensu Eukaryota). **Interpro** Ubiquitin.
CHS2 (YBR038W)	**SGD** Chitin synthase II, requires activation from zymogenic form in order to catalyze the transfer of N-acetylglucosamine (GlcNAc) to chitin; required for the synthesis of chitin in the primary septum during cytokinesis. **GO** chitin synthase activity. cytokinesis. bud neck. **Interpro** Chitin synthase; Glycosyl transferase, family 2.
PIG2 (Yil045wp)	**SGD** Putative type-1 protein phosphatase targeting subunit that tethers Glc7p type-1 protein phosphatase to Gsy2p glycogen synthase. **GO** cytoplasm. protein phosphatase type 1 regulator activity. regulation of glycogen biosynthetic process. **Interpro** This family consists of several eukaryotic proteins that are thought to be involved in the regulation of glycogen metabolism.
CWP1 (YKL096W)	**SGD** Cell wall mannoprotein, linked to a β-1,3- and β-1,6-glucan heteropolymer through a phosphodiester bond; involved in cell wall organization. **GO** structural constituent of cell wall. cell wall organization and biogenesis. cell wall. **Interpro** Yeast PIR protein repeat.
MNN1 (YER001W)	**SGD** α-1,3-mannosyltransferase, integral membrane glycoprotein of the Golgi complex, required for addition of α1,3-mannose linkages to N-linked and O-linked oligosaccharides, one of five *S. cerevisiae* proteins of the MNN1 family. **GO** α-1,3-mannosyltransferase activity. N-glycan processing; protein amino acid O-linked glycosylation. Golgi apparatus.
NCE102 (YPR149W)	**SGD** Protein of unknown function; contains transmembrane domains; involved in secretion of proteins that lack classical secretory signal sequences; component of the detergent-insoluble glycolipid-enriched complexes (DIGs). **GO** molecular function unknown. protein secretion. cytoplasm; endoplasmic reticulum; integral to membrane; mitochondrion.
DIP5 (YPL265W)	**SGD** Dicarboxylic amino acid permease, mediates high-affinity and high-capacity transport of L-glutamate and L-aspartate; also a transporter for Gln, Asn, Ser, Ala, and Gly. **GO** amino acid permease activity; amino acid transporter activity. amino acid transport. plasma membrane. **Interpro** Amino acid/polyamine transporter I; Amino acid permease; Amino acid permease-associated region; Yeast amino acid permease.
CDC5 (YMR001C)	**SGD** Polo-like kinase with similarity to *Xenopus* Plx1 and *S. pombe* Plo1p; found at bud neck, nucleus and SPBs; has multiple functions in mitosis and cytokinesis through phosphorylation of substrates; may be a Cdc28p substrate. **GO** protein serine/threonine kinase activity. DNA-dependent DNA replication; protein amino acid phosphorylation. Cellular Component: bud neck; nucleus; spindle pole. **Interpro** POLO box duplicated region; Protein kinase; Serine/threonine protein kinase, active site; Serine/threonine protein kinase.

Name	Description
SR04/AXL2 (YIL140W)	**SGD** Integral plasma membrane protein required for axial budding in haploid cells, localizes to the incipient bud site and bud neck; glycosylated by Pmt4p; potential Cdc28p substrate. **GO** molecular function unknown. axial bud site selection; bud site selection. bud neck; integral to plasma membrane; septin ring. **Interpro** Dystroglycan-type cadherin-like; Putative Ig - This alignment represents the conserved core region of a $\sim$ 90 residue repeat found in several haemagglutinins and other cell surface proteins.
HSL1 Ykl101w	**SGD** $Nim1p$-related protein kinase that regulates the morphogenesis and septin checkpoints; associates with the assembled septin filament; required along with $Hsl7p$ for bud neck recruitment, phosphorylation, and degradation of $Swe1p$ **GO** bud neck; septin ring. protein kinase activity. cell morphogenesis checkpoint; G2/M transition of mitotic cell cycle; protein amino acid phosphorylation; septin checkpoint. **Interpro** Protein kinases catalyze the phosphotransfer reaction fundamental to most signalling and regulatory processes in the eukaryotic cell.
CLN2 (YPL256C)	**SGD** G1 cyclin involved in regulation of the cell cycle; activates Cdc28p kinase to promote the G1 to S phase transition; late G1 specific expression depends on transcription factor complexes, MBF (Swi6p-Mbp1p) and SBF (Swi6p-Swi4p). **GO** cyclin-dependent protein kinase regulator activity. re-entry into mitotic cell cycle after pheromone arrest; regulation of cyclin dependent protein kinase activity. cytoplasm; nucleus. **Interpro** Cyclin, N-terminal; Cyclin.
SWE1 (YJL187C)	**SGD** Protein kinase that regulates the G2/M transition by inhibition of Cdc28p kinase activity; localizes to the nucleus and to the daughter side of the mother-bud neck; homolog of *S. pombe* Wee1p; potential Cdc28p substrate. **GO** protein kinase activity. G2/M transition of mitotic cell cycle; G2/M transition size control checkpoint; cell morphogenesis checkpoint; regulation of cyclin dependent protein kinase activity; regulation of meiosis. bud neck; nucleus. **Interpro** Serine/threonine protein kinase, active site; Serine/threonine protein kinase.
BEM1 (YBR200W)	**SGD** Protein containing SH3-domains, involved in establishing cell polarity and morphogenesis; functions as a scaffold protein for complexes that include Cdc24p, Ste5p, Ste20p, and Rsr1p. **GO** protein binding. cellular morphogenesis during conjugation with cellular fusion; establishment of cell polarity. bud neck; bud tip; incipient bud site; mating projection tip. **Interpro** Neutrophil cytosol factor 2; Octicosapeptide/Phox/Bem1p; Phox-like; SH3.
POL30 (YBR088C)	**SGD** Proliferating cell nuclear antigen (PCNA), functions as the sliding clamp for DNA polymerase δ; may function as a docking site for other proteins required for mitotic and meiotic chromosomal DNA replication and for DNA repair. **GO** DNA polymerase processivity factor activity. base-excision repair; lagging strand elongation; leading strand elongation; mismatch repair; mutagenesis; nucleotide-excision repair; postreplication repair. nucleus; replication fork. **Interpro** Proliferating cell nuclear antigen, PCNA.
